# The Relation Between Body Mass Index and Musculoskeletal Injury

**DOI:** 10.7759/cureus.28965

**Published:** 2022-09-09

**Authors:** Abdulaziz A Alangari, Mohammed M Almutairi, Abdulmalik M Alrrajeh, Mohammad A Aleidi, Mohammed A Alqarni, Hesham A Almeneif, Hamad K Alolaywi, Awad M Almuklass

**Affiliations:** 1 College of Medicine, King Saud Bin Abdulaziz University for Health Sciences College of Medicine, Riyadh, SAU; 2 Research, King Abdullah International Medical Research Center, Riyadh, SAU

**Keywords:** young adult, lower extremity, risk, bmi score, site of injury, musculoskeletal injury

## Abstract

Background

The nature and extent of the relation between body mass index (BMI) score and the risk of Musculoskeletal (MSK) injury are still unclear, with few studies investigating. So, the purpose of this study was to assess the association between BMI scores and MSK injury and to see if the site of MSK injury is affected by a specific BMI score. In addition, the risk of MSK injuries was compared among different adult age groups.

Methods

The study population included all patients above 18 years old with musculoskeletal injuries between January 2009 and December 2019 at King Abdulaziz Medical City (KAMC). The estimated sample size was 377. The study subjects were distributed according to their BMI into four categories (underweight, normal weight, overweight, and obese). Also, they were divided according to their age into young adults, middle age, and older adults. Each MSK injury was identified by its location as upper extremity, axial skeleton, or lower extremity.

Results

Only gender and age were significantly related to the site of injury, with P-values (0.018) and (0.001), respectively. As for the BMI category, its relation with the site of injury was nonsignificant (P-value: 0.092). The younger age group (≤ 35) has a significantly higher chance to be injured in the upper extremities compared with the older adults (≥ 56) (P-value = 0.014). While the axial skeleton (especially the lower back) was the most common site of injury in obese, overweight, and underweight categories, patients with normal BMI have lower extremities as their most common site of injury.

Conclusion

Although a higher BMI is associated with an increased risk of MSK injury, the difference in the BMI score seems to not effect the site of injury. By contrast, both gender and age group have a significant relationship with the site of MSK injury.

## Introduction

Musculoskeletal (MSK) injury refers to any injury affecting the muscle or skeletal system. This includes injuries to the bones, joints, ligaments, tendons, blood vessels, and peripheral nerves, which lead to discomfort, pain, or dysfunction [1]. There is a high prevalence of MSK injuries in the United States, with more than 6.8 million reported injuries that people sought medical treatment for, in 2012 only, most of these injuries affected people between 18 to 64 years old [2]. Gender, age, and physical fitness are factors that can be associated with the risk of MSK injuries. A study that was conducted on intensive care nurses showed a significant association between the female gender and musculoskeletal disorders [3]. Another study suggests that the risk of MSK injuries is approximately four- to five-fold higher for people whose age is ≥30 years old [4]. In addition, people who have high fitness levels have a lower risk compared with those who are less fit, and those with a BMI of more than 35 have a three times higher risk of MSK injury [5].

BMI is an acceptable measurement to determine physical fitness. Body mass index (BMI) is a formula that is used to assess the weight relative to the height of the person’s body. It is calculated by dividing the weight in kilogram (kg) by the height in meters squared. The ideal BMI for most adults is in the range of 18.5-24.9. While less than 18.5 is considered underweight, 25-29.9 is overweight, and more than 30 is obese [6].

Many diseases are associated with obesity or high BMI scores, such as type 2 diabetes mellitus, hypertension, and heart disease. On the other hand, the nature and extent of the relation between BMI score and MSK injury are still unclear, with few studies investigating it.

Some research studies showed an indirect correlation between BMI and the incidence of motor vehicle (MV) injuries. This is thought to be due to the decreased physical activity in high BMI individuals, so they rely more on motor vehicle transportation. Also, obese individual tends to avoid sports that need moderate to intense physical activity, so they have a lower rate of falls and sports-related injuries [7,8]. This is consistent with another study that suggested that physically active individuals have a 7% greater chance of sustaining an MSK injury [9].

Another study was conducted to examine the relation between BMI and the risk of occupational MSK injuries in the different job categories. They used 17 years of data from 38,214 university and healthcare workers. They found that there was a significant connection between BMI and the risk of occupational MSK injuries, especially in “low” risk occupations [10].

Also, one study was done to gather information about the frequency and type of injuries and illnesses among overweight and obese adults who engage in regular physical activities as part of weight loss or weight gain prevention programs. At six months intervals, participants were asked a question about any injury or illness affecting their exercise during the last six months. During 18 months of study, 46% reported at least one injury, and 32% reported at least one injury that was caused by exercise. Lower-body musculoskeletal injuries (21%) were the most common injury, followed by cold/flu/respiratory infections (18%) and back pain/injury (10%). Participants with higher BMI had a higher risk of injury over time than those with lower BMI [11].

A previous study was conducted on army soldiers who entered basic combat training. A variety of variables such as gender, age, weight, height, BMI, push-ups, 2-mile run times, and medical injury diagnosis were recorded. 147,398 men and 41,727 women whose data were collected in that study, and it showed increased injury risks as run times became slower. Groups of both genders that are average in BMI tend to have a lower risk of injury (average BMI with fastest run-times) with a significant relative risk compared to groups with the lowest BMI and slowest run-time. Most fit army trainees have a lower risk of injury related to training in general, while those with the lowest BMI tend to have a high risk of training-related injuries [12]. 

The effect of BMI on MSK injuries is still unclear, especially in the Saudi population. So, the purpose of this study is to assess the association of different BMI categories with the risk of MSK injury. Also, to show if the sites of MSK injuries were associated with specific BMI scores. In addition, to compare the risk of MSK injuries among different adult age groups.

## Materials and methods

A cross-sectional study was chosen for this research to determine if exposure to a specific risk factor (high BMI) might associate with the risk of MSK injury (type, location) in both genders. Simple random sampling was applied to the targeted population. The study population included all patients with musculoskeletal injuries between January 2015 and December 2019 at King Abdulaziz Medical City (KAMC) and excluded any patient who is younger than 18 years old or had a chronic disease that may cause MSK injury.

KAMC was established in May 1983 with a capacity of almost 1500 beds. It provides all levels of healthcare to the Ministry of National Guard personnel and their families, starting from primary healthcare to the most advanced tertiary services. Regarding the sample size, the total population was 20000, with a 95% confidence level and 5% margin of error. The estimated sample size was 377 patients.

This study was approved by King Abdullah International Medical Research Center (KAIMRC) (Memo Ref. No. IRBC/1939/20). The charts of all patients above 18 years old with MSK injuries, who visited KAMC from Jan 2009 to Dec 2019, were reviewed and checked for musculoskeletal injuries among different BMI categories. The research team collected the data from medical records in (BESTCare) system, then recorded and coded it on a Microsoft Excel (Redmond, USA) sheet. The main variables were gender, age, BMI, and the site of musculoskeletal injuries. The outcome variables were the nature of the relation between the BMI and the site of MSK injury. Also, the study assessed if gender or age group affects this relation.

The study subjects were distributed according to their BMI score into four categories. The first category includes patients with BMI under 18.5, the second is 18.6-24.9, the third is 25 -29.9, and the last one is 30 or more. In addition to that, they were divided into three age groups, younger adults (age 18-35 years), middle-aged (age 36-55 years), and older adults (older than 55 years). Lastly, Each MSK injury was identified by its location as upper extremity, axial skeleton, or lower extremity. 

For data analysis, we used IBM Corp. Released 2019. IBM SPSS Statistics for Windows, Version 26.0. Armonk, NY: IBM Corp. The baseline characteristics were presented as frequencies and percentages. A chi-square test was used to assess the association of the injury site with the age, gender, and BMI category. For the adjusted analysis, we used a multinomial logistic regression test. The test was considered significant if the P-value is ≤ 0.05.

## Results

This study included (377) participants, almost half of them were males (49.9%, N= 188), and most of them (35.3%) were younger than 36 years old. They were distributed according to the BMI score into four categories. The majority of the patients fall in the obese category (50.4%, N= 190), followed by overweight (31.8%, N= 120), then normal weight (14.9%, N= 56), and underweight (2.9%, N= 11) (Table 1). The most common site of injury was at the axial skeleton (49.9%, N= 188). (28.1%, N=106) of them have the injury at the lower extremity, and (22%, N= 83) have it at the upper extremity.

**Table 1:** Baseline characteristics.

		N	%
Gender	Female	189	50.1%
	Male	188	49.9%
Site Of Injury	Axial Skeleton	188	49.9%
	Lower Extremities	106	28.1%
	Upper Extremities	83	22.0%
Age	18 - 35	133	35.3%
	36 - 55	131	34.7%
	≥56	113	30.0%
BMI	Underweight	11	2.9%
	Normal	56	14.9%
	Overweight	120	31.8%
	Obese	190	50.4%

The unadjusted analyses that assess the association between gender, age and BMI with the site of injury are shown in (Table 2). Only gender and age were significantly related to the site of injury, with P-values (0.018) and (0.001), respectively (Table 2). As for the BMI category, its relationship with the site of injury was nonsignificant (P-value: 0.092). While the axial skeleton (especially the lower back) was the most common site of injury in obese, overweight, and underweight categories, patients with normal BMI have lower extremities as their most common site of injury. Regarding the age group, the axial injury was the most common in both middle age (59.5%, N= 78) and older adults (56.6%, N= 64), respectively (Table 2). In younger adults, the most common site was the lower extremity (37.6%, N= 50) (Table 2). 

**Table 2:** Unadjusted analysis using Chi-square test. The gender and age were significantly associated with the site of injury. * : Significant, p-value ≤ 0.05

Chi-square test								
		SITE OF INJURY						
		AXIAL		LOWER		UPPER		p-value
		Count	Row N%	Count	Row N%	Count	Row N%	
Gender	Female	108	57.10%	45	23.80%	36	19.00%	0.018 *
	Male	80	42.60%	61	32.40%	47	25.00%	
Age	18 - 35	46	34.60%	50	37.60%	37	27.80%	0.001 *
	36 - 55	78	59.50%	28	21.40%	25	19.10%	
	≥56	64	56.60%	28	24.80%	21	18.60%	
BMI	Underweight	6	54.50%	3	27.30%	2	18.20%	0.092
	Normal	18	32.10%	24	42.90%	14	25.00%	
	Overweight	60	50.00%	35	29.20%	25	20.80%	
	Obese	104	54.70%	44	23.20%	42	22.10%	

The adjusted analysis using a multinomial logistic regression test was done to understand whether MSK injury sites can be predicted based on gender, age, or BMI categories (Table 3). The main finding was that the younger age group (≤ 35) have a significantly higher chance to be injured in the upper extremities compared with the older adults (≥ 56) (P-value = 0.014), adjusted odd ratio (1.90) (Table 3). On the other hand, none of the four BMI categories shows a significant relationship with the specific site of injury. Also, in contrast to the unadjusted analysis, the gender here was nonsignificant as well. 

**Table 3:** Adjusted Analysis (multinomial logistic regression, reference category is: upper extremity injury). ^ : The control group. * : Significant, p-value ≤ 0.05. The young adults (age 18 to 35) are significantly at higher risk to have upper extremity injury compared with older adults ( ≥56 years old).

Parameter Estimates		Hypothesis Test	Exp(B)	95% Wald Confidence Interval for Exp(B)	
Parameter	B	Sig.		Lower	Upper
Threshold	Axial	0.924	1.02	0.63	1.66
	Lower	<0.001	3.88	2.34	6.42
Female		0.113	0.72	0.48	1.08
Male ^^^		.	1.00	.	.
Underweight		0.347	0.56	0.17	1.87
Normal Weight		0.325	1.33	0.75	2.35
Overweight		0.646	0.90	0.57	1.42
Obese ^^^		.	1.00	.	.
18 - 35		0.014 *	1.90	1.14	3.17
36 - 55		0.663	0.90	0.54	1.48
≥56 ^^^		.	1.00	.	.
Dependent Variable: SITE OF INJURY					
Model: (Threshold), GENDER, BMI, Age					
b Fixed at the displayed value.					

## Discussion

This study investigated the nature of the relationship between BMI and the site of MSK injuries in both males and females among three different age groups (young adults, middle age, and older adults). As shown in the result, most of the patients with MSK injury were obese (50.4%) or overweight (31.8%). This is consistent with a previous study, where they found that increased BMI is significantly associated with the risk of injury in the lower back [1]. The association of the risk of developing lower back pain with a higher BMI score has been thoroughly studied. It investigated the lumbosacral angles in individuals with a higher BMI, which leads to a biomechanical change in the lumbosacral spine, which increase the incidence of low back pain [13]. This could be due to the higher pressure exerted on the joints and ligaments by the body mass.

However, our results seem to suggest that the difference in the BMI category does not influence the site of injury. This is incongruous with a previous study where the BMI was a statistically significant predictor of MSK injury at the lower extremity among men but not among women [14]. Compared to our study, they had a much greater sample size, so it is possible that with larger sample size, the numbers could reach statistical significance.

On the other hand, both the gender and age group have a significant effect on the site of injury in the unadjusted analysis. After the adjusted analysis, only the age shows significant relations where the younger adults have a higher chance to be injured at the upper extremity compared with the older adult group (P-value = 0.014). This could be attributed to the fact that upper extremity injury is usually linked to sports which are more commonly practiced by younger people. This is supported by a previous work that pointed out that young age is one of the risk factors for upper extremity injuries along with playing individual sports [15].

The main limitation here is the possible hospital admission bias since there was no information on using an objective scale to assess the injury. Also, interviewing the participants was not possible. This prevented gathering other related information that might have an impact on the result, like their lifestyle, level of physical activity, the cause of injury, etc. So further studies that include other co-factors and with a larger sample size are suggested to reach the most accurate outcome regarding the BMI-MSK injury relationship.

## Conclusions

Although a higher BMI is associated with an increased risk of MSK injury, the difference in the BMI score seems to have no effect on the site of injury. By contrast, both gender and age group have a significant relationship with the site of MSK injury. In addition to that, younger adults (≤ 35 years old) are more prone to be injured in the upper extremities compared with older adults (≥ 56 years old). This is most likely due to the connection between this type of injury and the sports that young adults usually practice.
